# Supplementary material for: COVID-19 Subunit Vaccine with a Combination of TLR1/2 and TLR3 Agonists Induces Robust and Protective Immunity
Source: Vaccines (Basel). 2021 Aug 27;9(9):957. doi: 10.3390/vaccines9090957 (PMC8473206; doi:10.3390/vaccines9090957)
Supplement: Supplementary file 1 [file vaccines-09-00957-s001.zip › vaccines-1325737-supplementary.pdf]

Supplementary Mateiral

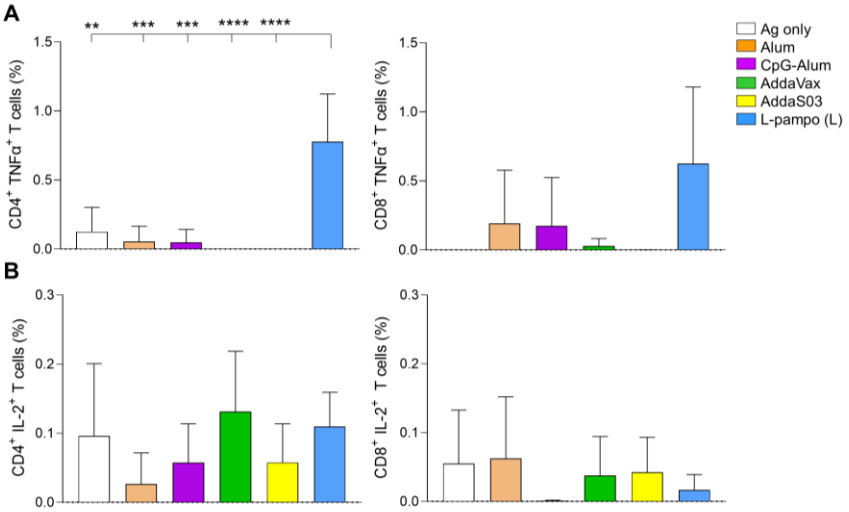

Figure S1. L-pampo increases TNF-α-producing T cells. Related to Figure 3. (A-B) BALB/c mice (total n=42; n=6/antigen only; n=8/Alum, AddaVax, AddaS03 or L-pampo(L); n=4/CpG-Alum) (n=4-8/group) were immunized with RBD-Fc with or without adjuvants i.m. on day 0 and day 21 as described in Figure 3A. At day 35, splenocytes were stimulated with PepMix™ SARS-CoV-2 (S-RBD) peptide pool and analyzed TNF-α or IL-2-producing $CD4^+$ or $CD8^+$ T cells by using intracellular cytokine assay. (A) Percentage of TNF-α-producing $CD4^+$ or $CD8^+$ T cells. (B) Percentage of IL-2-producing $CD4^+$ or $CD8^+$ T cells. Data shown are mean ± SEM. Data reflect 2 independent experiments. Asterisks indicates statistically significant differences in comparison to the L-pampo (L). **$p < 0.01$, ***$p < 0.001$, ****$p < 0.0001$, one way ANOVA with Tukey's test.

Table S1. Detailed values of each group in Figures 1D and 2A-2B.

	Group	Sample1	Sample2	Sample3	Sample4	Sample5	Sample6	Sample7	Sample8
**Figure 1D**	Ag only	164.5	0.0	9471.9	316.8	668.8	610.2	0.0	5300.0
**(Total Ab)**	Alum	15015.6	617.2	0.0	0.0	6284.4	0.0	264.1	938.3
(GMT)	AddaVax	126718.8	87968.8	68046.9	112656.3	136093.8	356562.5	21171.9	87968.8
	AddaS03	328437.5	33437.5	102031.3	73906.3	12671.9	173593.8	157187.5	85625.0
	L-pampo (L)	694375.0	75078.1	194687.5	328437.5	328437.5	24875.0	276875.0	98515.6
	L-pampo (H)	133750.0	380000.0	276875.0	79765.6	230000.0	290937.5	143125.0	171250.0
**Figure 1D**	Ag only	223.0	100.0	200.0	610.2	2768.8	4784.4	996.9	16187.5
**(RBD IgG1)**	Alum	19703.1	164.5	200.0	250.0	7625.0	123.4	1173.4	4456.3
(GMT)	AddaVax	287500.0	181835.9	161328.1	275781.3	488125.0	1416250.0	68046.9	290937.5
	AddaS03	563281.3	68945.3	255078.1	137890.6	50468.8	441250.0	516250.0	244062.5
	L-pampo (L)	973437.5	16132.8	145468.8	403281.3	124375.0	75078.1	544375.0	262812.5
	L-pampo (H)	234531.3	262656.3	567343.8	36953.1	124375.0	1013125.0	143125.0	610000.0
**Figure 1D**	Ag only	0.0	0.0	410.5	0.0	0.0	0.0	0.0	0.0
**(RBD IgG2a)**	Alum	2346.9	0.0	0.0	0.0	0.0	0.0	0.0	0.0
(GMT)	AddaVax	6425.0	1008.6	1043.8	2206.3	4515.6	4457.0	1937.5	680.5
	AddaS03	33312.5	346.1	1314.1	2721.9	328.5	3460.9	3285.2	868.0
	L-pampo (L)	89125.0	52562.5	110687.5	91000.0	97531.3	2406.3	108312.5	5984.4
	L-pampo (H)	10320.3	73187.5	42250.0	11500.0	86796.9	19703.1	97062.5	104093.8
**Figure 1D**	Ag only	0.0	0.0	645.3	0.0	0.0	0.0	0.0	246.5
**(RBD IgG2b)**	Alum	2956.3	0.0	0.0	0.0	246.5	0.0	463.3	0.0
(GMT)	AddaVax	29921.9	1103.1	2253.1	6171.9	6284.4	10221.9	310.9	1571.9
	AddaS03	70390.6	1829.7	2768.8	15253.9	269.9	6171.9	6757.8	5585.9
	L-pampo (L)	96171.9	16718.8	59843.8	140781.3	100859.4	1454.7	78593.8	10859.4
	L-pampo (H)	20527.3	56328.1	119687.5	21699.2	85625.0	53984.4	36367.2	26992.2
**Figure 2A**	Ag only	16.0	10.0	3.0	15.0	4.0	2.0	5.0	4.0
**(IFN-γ SFCs)**	Alum	21.0	7.0	10.0	13.0	11.0	6.0	14.0	12.0
(No.)	AddaVax	15.0	34.0	25.0	19.0	30.0	63.0	16.0	16.0
	AddaS03	39.0	13.0	29.0	17.0	21.0	19.0	17.0	10.0
	L-pampo (L)	220.0	38.0	44.0	77.0	80.0	63.0	108.0	62.0
	L-pampo (H)	31.0	42.0	151.0	47.0	225.0	57.0	149.0	60.0
**Figure 2B**	Ag only	650.8	923.8	634.0	783.3	492.0	975.0	1052.3	
**((IFN-γ)**	Alum	631.5	845.3	542.8	2614.0	2299.0	2198.0	1324.7	
(pg/ml)	AddaVax	1218.8	4665.3	3660.0	5523.0	3785.7	3597.7	3849.6	
	AddaS03	3732.8	4418.5	2646.8	2291.3	2935.7	2528.3	3932.3	
	L-pampo (L)	11975.0	11206.3	10762.8	7886.0	10782.7	5742.0	4109.0	
	L-pampo (H)	8188.3	14719.3	14482.3	12433.7	7919.3	6473.3	8068.3	

Table S2. Detailed values of each group in Figures 3D and 4A-4B.

	Group	Sample1	Sample2	Sample3	Sample4	Sample5	Sample6	Sample7	Sample8
**Figure 3D**	Ag only	2251.6	3190.6	252.3	0.0	269.9	439.8		
**(Total Ab)**	Alum	9851.6	10796.9	9500.0	6570.3	668.8	4457.0	0.0	586.7
(GMT)	CpG-Alum	80937.5	112656.3	89140.6	79765.6				
	AddaVax	52812.5	12343.8	82109.4	63359.4	71562.5	86796.9	28156.3	70390.6
	AddaS03	11757.8	22890.6	21718.8	21718.8	72687.5	110187.5	40812.5	17593.8
	L-pampo (L)	39296.9	35781.3	43398.4	26992.2	147812.5	112656.3	71562.5	26406.3
**Figure 3D**	Ag only	0.0	0.0	504.3	0.0	123.4	1150.0		
**(RBD IgG1)**	Alum	6101.6	7742.2	8328.1	4398.4	926.6	7268.8	0.0	1126.6
(GMT)	CpG-Alum	79765.6	117343.8	80937.5	70390.6				
	AddaVax	41054.7	10585.9	78593.8	72734.4	102031.3	136093.8	65703.1	92656.3
	AddaS03	11171.9	22890.6	24648.4	26992.2	107375.0	102031.3	87687.5	52062.5
	L-pampo (L)	24648.4	18203.1	58671.9	51601.6	102031.3	122031.3	119687.5	43984.4
**Figure 3D**	Ag only	0.0	0.0	0.0	0.0	0.0	0.0		
**(RBD IgG2a)**	Alum	135.2	199.6	170.3	158.6	0.0	0.0	0.0	0.0
(GMT)	CpG-Alum	13609.4	6101.6	10203.1	16187.5				
	AddaVax	7625.0	428.1	1234.4	1585.9	4632.8	2406.3	486.7	539.5
	AddaS03	228.9	516.0	457.4	357.8	1220.3	926.6	668.8	504.3
	L-pampo (L)	6101.6	9265.6	4632.8	4925.8	34718.8	9148.4	13375.0	6921.9
**Figure 3D**	Ag only	0.0	0.0	0.0	0.0	0.0	0.0		
**(RBD IgG2b)**	Alum	141.0	123.4	158.6	117.6	0.0	146.9	0.0	0.0
(GMT)	CpG-Alum	6921.9	5394.5	4046.9	6687.5				
	AddaVax	9031.3	645.3	3168.0	1996.1	4695.3	2585.9	868.0	727.3
	AddaS03	381.3	832.8	680.5	938.3	1320.3	1261.7	1203.1	793.0
	L-pampo (L)	3285.2	5101.6	4281.3	2640.6	8562.5	11031.3	9382.8	7742.2
**Figure 4A**	Ag only	14.0	22.0	3.0	11.0	7.0	4.0		
**(IFN-γ SFCs)**	Alum	39.0	35.0	37.0	10.0	0.0	5.0	17.0	6.0
(No.)	CpG-Alum	37.0	52.0	69.0	50.0				
	AddaVax	54.0	40.0	32.0	27.0	45.0	17.0	42.0	2.0
	AddaS03	2.0	22.0	34.0	37.0	33.0	14.0	20.0	10.0
	L-pampo (L)	32.0	62.0	70.0	69.0	82.0	46.0	82.0	18.0
**Figure 4B**	Ag only	305.8	479.0	331.0	291.3	435.8	240.2	1052.3	
**((IFN-γ)**	Alum	866.0	723.3	720.0	136.5	240.5	288.9	463.2	328.5
(pg/ml)	CpG-Alum	2201.0	2914.5	3160.3	2215.3				
	AddaVax	1533.5	795.0	676.0	694.8	1781.1	969.0	1382.8	830.8
	AddaS03	497.9	937.3	1074.3	990.3	2853.2	843.8	1262.8	626.3
	L-pampo (L)	1586.8	2469.5	2818.3	2994.8	8062.1	2665.3	7742.3	2853.7
